# Supplementary figures and images for: The proliferation of antibiotic resistance genes (ARGs) and microbial communities in industrial wastewater treatment plant treating N,N-dimethylformamide (DMF) by AAO process
Source: PLoS One. 2024 Apr 10;19(4):e0299740. doi: 10.1371/journal.pone.0299740 (PMC11006197; doi:10.1371/journal.pone.0299740)

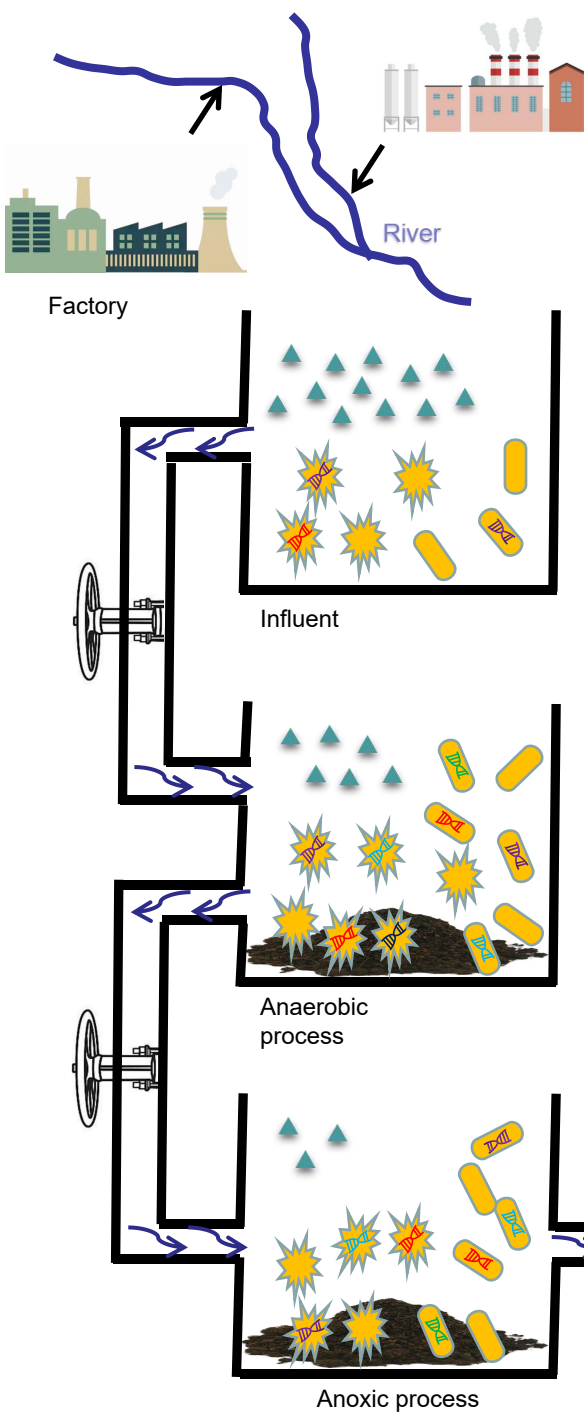

Changes of ARGs, bacterial community and DMF in AOA process

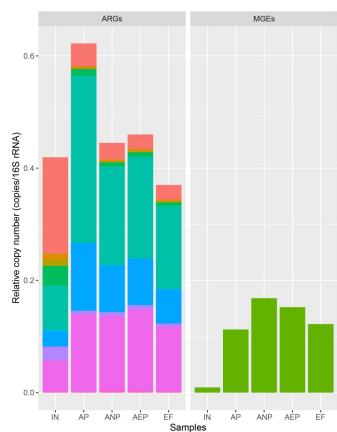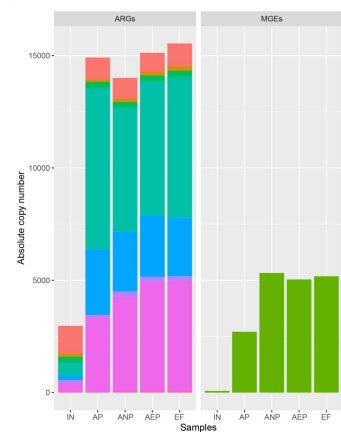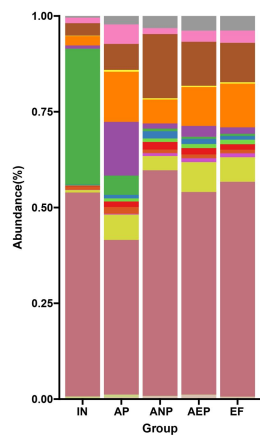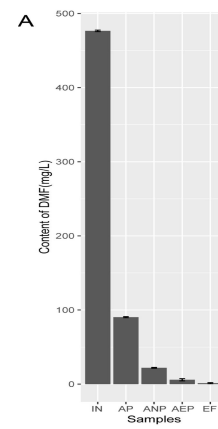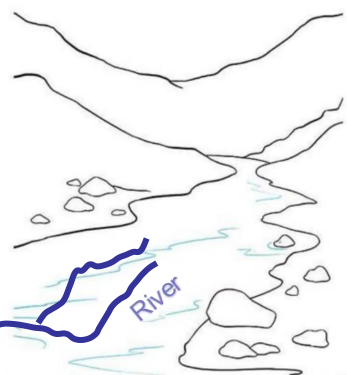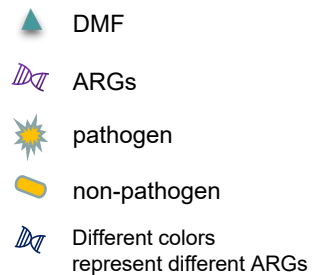

Supplement: S1 Fig — (PDF) [file pone.0299740.s001.pdf]
