# Supplementary material for: The proliferation of antibiotic resistance genes (ARGs) and microbial communities in industrial wastewater treatment plant treating N,N-dimethylformamide (DMF) by AAO process
Source: PLoS One. 2024 Apr 10;19(4):e0299740. doi: 10.1371/journal.pone.0299740 (PMC11006197; doi:10.1371/journal.pone.0299740)
Supplement: S1 Table — (DOCX) [file pone.0299740.s002.docx]

Table S1 primers used for ARGs detection and 16S rRNA sequencing

| **NO.** | **Gene Name** | **Forward Primer** | **Reverse Primer** |
| --- | --- | --- | --- |
| 1 | aac | CCCTGCGTTGTGGCTATGT | TTGGCCACGCCAATCC |
| 2 | aac(6')I1 | GACCGGATTAAGGCCGATG | CTTGCCTTGATATTCAGTTTTTATAACCA |
| 3 | aac(6')-Ib(aka aacA4)-01 | GTTTGAGAGGCAAGGTACCGTAA | GAATGCCTGGCGTGTTTGA |
| 4 | aac(6')-Ib(aka aacA4)-02 | CGTCGCCGAGCAACTTG | CGGTACCTTGCCTCTCAAACC |
| 5 | aac(6')-Ib(aka aacA4)-03 | AGAAGCACGCCCGACACTT | GCTCTCCATTCAGCATTGCA |
| 6 | aac(6')-II | CGACCCGACTCCGAACAA | GCACGAATCCTGCCTTCTCA |
| 7 | aac(6')-Iy | GCTTTGCGGATGCCTCAAT | GGAGAACAAAAATACCTTCAAGGAAA |
| 8 | aacA/aphD | AGAGCCTTGGGAAGATGAAGTTT | TTGATCCATACCATAGACTATCTCATCA |
| 9 | aacC | CGTCACTTATTCGATGCCCTTAC | GTCGGGCGCGGCATA |
| 10 | aacC1 | GGTCGTGAGTTCGGAGACGTA | GCAAGTTCCCGAGGTAATCG |
| 11 | aacC2 | ACGGCATTCTCGATTGCTTT | CCGAGCTTCACGTAAGCATTT |
| 12 | aacC4 | CGGCGTGGGACACGAT | AGGGAACCTTTGCCATCAACT |
| 13 | aadA-01 | GTTGTGCACGACGACATCATT | GGCTCGAAGATACCTGCAAGAA |
| 14 | aadA-02 | CGAGATTCTCCGCGCTGTA | GCTGCCATTCTCCAAATTGC |
| 15 | aadA1 | AGCTAAGCGCGAACTGCAAT | TGGCTCGAAGATACCTGCAA |
| 16 | aadA-1-01 | AAAAGCCCGAAGAGGAACTTG | CATCTTTCACAAAGATGTTGCTGTCT |
| 17 | aadA-1-02 | CGGAATTGAAAAAACTGATCGAA | ATACCGGCTGTCCGTCATTT |
| 18 | aadA2-01 | ACGGCTCCGCAGTGGAT | GGCCACAGTAACCAACAAATCA |
| 19 | aadA2-02 | CTTGTCGTGCATGACGACATC | TCGAAGATACCCGCAAGAATG |
| 20 | aadA2-03 | CAATGACATTCTTGCGGGTATC | GACCTACCAAGGCAACGCTATG |
| 21 | aadA5-01 | ATCACGATCTTGCGATTTTGCT | CTGCGGATGGGCCTAGAAG |
| 22 | aadA5-02 | GTTCTTGCTCTTGCTCGCATT | GATGCTCGGCAGGCAAAC |
| 23 | aadA9-01 | CGCGGCAAGCCTATCTTG | CAAATCAGCGACCGCAGACT |
| 24 | aadA9-02 | GGATGCACGCTTGGATGAA | CCTCTAGCGGCCGGAGTATT |
| 25 | aadD | CCGACAACATTTCTACCATCCTT | ACCGAAGCGCTCGTCGTATA |
| 26 | aadE | TACCTTATTGCCCTTGGAAGAGTTA | GGAACTATGTCCCTTTTAATTCTACAATCT |
| 27 | acrA-01 | CAACGATCGGACGGGTTTC | TGGCGATGCCACCGTACT |
| 28 | acrA-02 | GGTCTATCACCCTACGCGCTATC | GCGCGCACGAACATACC |
| 29 | acrA-03 | CAGACCCGCATCGCATATT | CGACAATTTCGCGCTCATG |
| 30 | acrA-04 | TACTTTGCGCGCCATCTTC | CGTGCGCGAACGAACAT |
| 31 | acrA-05 | CGTGCGCGAACGAACA | ACTTTGCGCGCCATCTTC |
| 32 | acrB-01 | AGTCGGTGTTCGCCGTTAAC | CAAGGAAACGAACGCAATACC |
| 33 | acrF | GCGGCCAGGCACAAAA | TACGCTCTTCCCACGGTTTC |
| 34 | acrR-01 | GCGCTGGAGACACGACAAC | GCCTTGCTGCGAGAACAAA |
| 35 | acrR-02 | GATGATACCCCCTGCTGTGAGA | ACCAAACAAGAAGCGCAAGAA |
| 36 | adeA | CAGTTCGAGCGCCTATTTCTG | CGCCCTGACCGACCAAT |
| 37 | ampC/blaDHA | TGGCCGCAGCAGAAAGA | CCGTTTTATGCACCCAGGAA |
| 38 | ampC-01 | TGGCGTATCGGGTCAATGT | CTCCACGGGCCAGTTGAG |
| 39 | ampC-02 | GCAGCACGCCCCGTAA | TGTACCCATGATGCGCGTACT |
| 40 | ampC-04 | TCCGGTGACGCGACAGA | CAGCACGCCGGTGAAAGT |
| 41 | ampC-05 | CTGTTCGAGCTGGGTTCTATAAGTAAA | CAGTATCTGGTCACCGGATCGT |
| 42 | ampC-06 | CCGCTCAAGCTGGACCATAC | CCATATCCTGCACGTTGGTTT |
| 43 | ampC-07 | CCGCCCAGAGCAAGGACTA | GCTCGACTTCACGCCGTAAG |
| 44 | ampC-09 | CAGCCGCTGATGAAAAAATATG | CAGCGAGCCCACTTCGA |
| 45 | aph | TTTCAGCAAGTGGATCATGTTAAAAT | CCAAGCTGTTTCCACTGTTTTTC |
| 46 | aph(2')-Id-01 | TGAGCAGTATCATAAGTTGAGTGAAAAG | GACAGAACAATCAATCTCTATGGAATG |
| 47 | aph(2')-Id-02 | TAAGGATATACCGACAGTTTTGGAAA | TTTAATCCCTCTTCATACCAATCCATA |
| 48 | aph6ia | CCCATCCCATGTGTAAGGAAA | GCCACCGCTTCTGCTGTAC |
| 49 | aphA1(aka kanR) | TGAACAAGTCTGGAAAGAAATGCA | CCTATTAATTTCCCCTCGTCAAAAA |
| 50 | bacA-01 | CGGCTTCGTGACCTCGTT | ACAATGCGATACCAGGCAAAT |
| 51 | bacA-02 | TTCCACGACACGATTAAGTCATTG | CGGCTCTTTCGGCTTCAG |
| 52 | bla1 | GCAAGTTGAAGCGAAAGAAAAGA | TACCAGTATCAATCGCATATACACCTAA |
| 53 | bla-ACC-1 | CACACAGCTGATGGCTTATCTAAAA | AATAAACGCGATGGGTTCCA |
| 54 | blaCMY | CCGCGGCGAAATTAAGC | GCCACTGTTTGCCTGTCAGTT |
| 55 | blaCMY2-01 | AAAGCCTCAT GGGTGCATAAA | ATAGCTTTTGTTTGCCAGCATCA |
| 56 | blaCMY2-02 | GCGAGCAGCCTGAAGCA | CGGATGGGCTTGTCCTCTT |
| 57 | blaCTX-M-01 | GGAGGCGTGACGGCTTTT | TTCAGTGCGATCCAGACGAA |
| 58 | blaCTX-M-02 | GCCGCGGTGCTGAAGA | ATCGGATTATAGTTAACCAGGTCAGATTT |
| 59 | blaCTX-M-03 | CGATACCACCACGCCGTTA | GCATTGCCCAACGTCAGATT |
| 60 | blaCTX-M-04 | CTTGGCGTTGCGCTGAT | CGTTCATCGGCACGGTAGA |
| 61 | blaCTX-M-05 | GCGATAACGTGGCGATGAAT | GTCGAGACGGAACGTTTCGT |
| 62 | blaCTX-M-06 | CACAGTTGGTGACGTGGCTTAA | CTCCGCTGCCGGTTTTATC |
| 63 | blaGES | GCAATGTGCTCAACGTTCAAG | GTGCCTGAGTCAATTCTTTCAAAG |
| 64 | blaIMP-01 | AACACGGTTTGGTGGTTCTTGTA | GCGCTCCACAAACCAATTG |
| 65 | blaIMP-02 | AAGGCAGCATTTCCTCTCATTTT | GGATAGATCGAGAATTAAGCCACTCT |
| 66 | bla-L1 | CACCGGGTTACCAGCTGAAG | GCGAAGCTGCGCTTGTAGTC |
| 67 | blaMOX/blaCMY | CTATGTCAATGTGCCGAAGCA | GGCTTGTCCTCTTTCGAATAGC |
| 68 | blaOCH | GGCGACTTGCGCCGTAT | TTTTCTGCTCGGCCATGAG |
| 69 | blaOKP | GCCGCCATCACCATGAG | GGTGACGTTGTCACCGATCTG |
| 70 | blaOXA1/blaOXA30 | CGGATGGTTTGAAGGGTTTATTAT | TCTTGGCTTTTATGCTTGATGTTAA |
| 71 | blaOXA10-01 | CGCAATTATCGGCCTAGAAACT | TTGGCTTTCCGTCCCATTT |
| 72 | blaOXA10-02 | CGCAATTATCGGCCTAGAAACT | TTGGCTTTCCGTCCCATTT |
| 73 | blaOXY | CGTTCAGGCGGCAGGTT | GCCGCGATATAAGATTTGAGAATT |
| 74 | blaPAO | CGCCGTACAACCGGTGAT | GAAGTAATGCGGTTCTCCTTTCA |
| 75 | blaPER | TGCTGGTTGCTGTTTTTGTGA | CCTGCGCAATGATAGCTTCAT |
| 76 | blaPSE | TTGTGACCTATTCCCCTGTAATAGAA | TGCGAAGCACGCATCATC |
| 77 | blaROB | GCAAAGGCATGACGATTGC | CGCGCTGTTGTCGCTAAA |
| 78 | blaSFO | CCGCCGCCATCCAGTA | GGGCCGCCAAGATGCT |
| 79 | blaSHV-01 | TCCCATGATGAGCACCTTTAAA | TTCGTCACCGGCATCCA |
| 80 | blaSHV-02 | CTTTCCCATGATGAGCACCTTT | TCCTGCTGGCGATAGTGGAT |
| 81 | blaTEM | AGCATCTTACGGATGGCATGA | TCCTCCGATCGTTGTCAGAAGT |
| 82 | blaTLA | ACACTTTGCCATTGCTGTTTATGT | TGCAAATTTCGGCAATAATCTTT |
| 83 | blaVEB | CCCGATGCAAAGCGTTATG | GAAAGATTCCCTTTATCTATCTCAGACAA |
| 84 | blaVIM | GCACTTCTCGCGGAGATTG | CGACGGTGATGCGTACGTT |
| 85 | blaZ | GGAGATAAAGTAACAAATCCAGTTAGATATGA | TGCTTAATTTTCCATTTGCGATAAG |
| 86 | carB | GGAGTGAGGCTGACCGTAGAAG | TGCTTAATTTTCCATTTGCGATAAG |
| 87 | catA1 | GGGTGAGTTTCACCAGTTTTGATT | CACCTTGTCGCCTTGCGTATA |
| 88 | catB3 | GCACTCGATGCCTTCCAAAA | AGAGCCGATCCAAACGTCAT |
| 89 | catB8 | CACTCGACGCCTTCCAAAG | CCGAGCCTATCCAGACATCATT |
| 90 | ceoA | ATCAACACGGACCAGGACAAG | GGAAAGTCCGCTCACGATGA |
| 91 | cepA | AGTTGCGCAGAACAGTCCTCTT | TCGTATCTTGCCCGTCGATAAT |
| 92 | cfiA | GCAGCGTTGCTGGACACA | GTTCGGGATAAACGTGGTGACT |
| 93 | cfr | GCAAAATTCAGAGCAAGTTACGAA | AAAATGACTCCCAACCTGCTTTAT |
| 94 | cfxA | TCATTCCTCGTTCAAGTTTTCAGA | TGCAGCACCAAGAGGAGATGT |
| 95 | intI-1(clinic) | GGCATCCAAGCAGCAAG | AAGCAGACTTGACCTGA |
| 96 | cmeA | GCAGCAAAGAAGAAGCACCAA | AGCAGGGTAAGTAAAACTAAGTGGTAAATCT |
| 97 | cmlA1-01 | TAGGAAGCATCGGAACGTTGAT | CAGACCGAGCACGACTGTTG |
| 98 | cmlA1-02 | AGGAAGCATCGGAACGTTGA | ACAGACCGAGCACGACTGTTG |
| 99 | cmr | CGGCATCGTCAGTGGAATT | CGGTTCCGAAAAAGATGGAA |
| 100 | cmx(A) | GCGATCGCCATCCTCTGT | TCGACACGGAGCCTTGGT |
| 101 | cphA-01 | GCGAGCTGCACAAGCTGAT | CGGCCCAGTCGCTCTTC |
| 102 | cphA-02 | GTGCTGATGGCGAGTTTCTG | GGTGTGGTAGTTGGTGTTGATCAC |
| 103 | dfrA1 | GGAATGGCCCTGATATTCCA | AGTCTTGCGTCCAACCAACAG |
| 104 | dfrA12 | CCTCTACCGAACCGTCACACA | GCGACAGCGTTGAAACAACTAC |
| 105 | emrD | CTCAGCAGTATGGTGGTAAGCATT | ACCAGGCGCCGAAGAAC |
| 106 | ereA | CCTGTGGTACGGAGAATTCATGT | ACCGCATTCGCTTTGCTT |
| 107 | ereB | GCTTTATTTCAGGAGGCGGAAT | TTTTAAATGCCACAGCACAGAATC |
| 108 | erm(34) | GCGCGTTGACGACGATTT | TGGTCATACTCGACGGCTAGAAC |
| 109 | erm(35) | TTGAAAACGATGTTGCATTAAGTCA | TCTATAATCACAACTAACCACTTGAACGT |
| 110 | erm(36) | GGCGGACCGACTTGCAT | TCTGCGTTGACGACGGTTAC |
| 111 | ermA | TTGAGAAGGGATTTGCGAAAAG | ATATCCATCTCCACCATTAATAGTAAACC |
| 112 | ermA/ermTR | ACATTTTACCAAGGAACTTGTGGAA | GTGGCATGACATAAACCTTCATCA |
| 113 | ermB | TAAAGGGCATTTAACGACGAAACT | TTTATACCTCTGTTTGTTAGGGAATTGAA |
| 114 | ermC | TTTGAAATCGGCTCAGGAAAA | ATGGTCTATTTCAATGGCAGTTACG |
| 115 | ermF | CAGCTTTGGTTGAACATTTACGAA | AAATTCCTAAAATCACAACCGACAA |
| 116 | ermJ/ermD | GGACTCGGCAATGGTCAGAA | CCCCGAAACGCAATATAATGTT |
| 117 | ermK-01 | GTTTGATATTGGCATTGTCAGAGAAA | ACCATTGCCGAGTCCACTTT |
| 118 | ermK-02 | GAGCCGCAAGCCCCTTT | GTGTTTCATTTGACGCGGAGTAA |
| 119 | ermT-01 | GTTCACTAGCACTATTTTTAATGACAGAAGT | GAAGGGTGTCTTTTTAATACAATTAACGA |
| 120 | ermT-02 | GTAAAATCCCTAGAGAATACTTTCATCCA | TGAGTGATATTTTTGAAGGGTGTCTT |
| 121 | ermX | GCTCAGTGGTCCCCATGGT | ATCCCCCCGTCAACGTTT |
| 122 | ermY | TTGTCTTTGAAAGTGAAGCAACAGT | TAACGCTAGAGAACGATTTGTATTGAG |
| 123 | fabK | TTTCAGCTCAGCACTTTGGTCAT | AAGGCATCTTTTTCAGCCAGTTC |
| 124 | floR | ATTGTCTTCACGGTGTCCGTTA | CCGCGATGTCGTCGAACT |
| 125 | folA | CGAGCAGTTCCTGCCAAAG | CCCAGTCATCCGGTTCATAATC |
| 126 | fosB | TCACTGTAACTAATGAAGCATTAGACCAT | CCATCTGGATCTGTAAAGTAAAGAGATC |
| 127 | fosX | GATTAAGCCATATCACTTTAATTGTGAAAG | TCTCCTTCCATAATGCAAATCCA |
| 128 | fox5 | GGTTTGCCGCTGCAGTTC | GCGGCCAGGTGACCAA |
| 129 | imiR | CCGGACTAGAGCTTCATGTAAGC | CCCACGCGGTACTCTTGTAAA |
| 130 | cIntI-1(class1) | CGAACGAGTGGCGGAGGGTG | TACCCGAGAGCTTGGCACCCA |
| 131 | IS613 | AGGTTCGGACTCAATGCAACA | TTCAGCACATACCGCCTTGAT |
| 132 | lmrA-01 | TCGACGTGACCGTAGTGAACA | CGTGACTACCCAGGTGAGTTGA |
| 133 | lnuA-01 | TGACGCTCAACACACTCAAAAA | TTCATGCTTAAGTTCCATACGTGAA |
| 134 | lnuB-01 | TGAACATAATCCCCTCGTTTAAAGAT | TAATTGCCCTGTTTCATCGTAAATAA |
| 135 | lnuB-02 | AAAGGAGAAGGTGACCAATACTCTGA | GGAGCTACGTCAAACAACCAGTT |
| 136 | lnuC | TGGTCAATATAACAGATGTAAACCAGATTT | CACCCCAGCCACCATCAA |
| 137 | marR-01 | GCGGCGTACTGGTGAAGCTA | TGCCCTGGTCGTTGATGA |
| 138 | matA/mel | TAGTAGGCAAGCTCGGTGTTGA | CCTGTGCTATTTTAAGCCTTGTTTCT |
| 139 | mdetl1 | ATACAGCAGTGGATATTGGTTTAATTGT | TGCATAAGGTGAATGTTCCATGA |
| 140 | mdtA | CCTAACGGGCGTGACTTCA | TTCACCTGTTTCAAGGGTCAAA |
| 141 | mdtE/yhiU | CGTCGGCGCACTCGTT | TCCAGACGTTGTACGGTAACCA |
| 142 | mecA | GGTTACGGACAAGGTGAAATACTGAT | TGTCTTTTAATAAGTGAGGTGCGTTAATA |
| 143 | mefA | CCGTAGCATTGGAACAGCTTTT | AAACGGAGTATAAGAGTGCTGCAA |
| 144 | mepA | ATCGGTCGCTCTTCGTTCAC | ATAAATAGGATCGAGCTGCTGGAT |
| 145 | mexA | AGGACAACGCTATGCAACGAA | CCGGAAAGGGCCGAAAT |
| 146 | mexD | TTGCCACTGGCTTTCATGAG | CACTGCGGAGAACTGTCTGTAGA |
| 147 | mexE | GGTCAGCACCGACAAGGTCTAC | AGCTCGACGTACTTGAGGAACAC |
| 148 | mexF | CCGCGAGAAGGCCAAGA | TTGAGTTCGGCGGTGATGA |
| 149 | mphA-01 | CTGACGCGCTCCGTGTT | GGTGGTGCATGGCGATCT |
| 150 | mphA-02 | TGATGACCCTGCCATCGA | TTCGCGAGCCCCTCTTC |
| 151 | mphB | CGCAGCGCTTGATCTTGTAG | TTACTGCATCCATACGCTGCTT |
| 152 | mphC | CGTTTGAAGTACCGAATTGGAAA | GCTGCGGGTTTGCCTGTA |
| 153 | msrA-01 | CTGCTAACACAAGTACGATTCCAAAT | TCAAGTAAAGTTGTCTTACCTACACCATT |
| 154 | msrC-01 | TCAGACCGGATCGGTTGTC | CCTATTTTTTGGAGTCTTCTCTCTAATGTT |
| 155 | mtrC-01 | GGACGGGAAGATGGTCCAA | CGTAGCGTTCCGGTTCGAT |
| 156 | mtrC-02 | CGGAGTCCATCGACCATTTG | ATCGTCGGCAAGGAGAATCA |
| 157 | mtrD-02 | GGTCGGCACGCTCTTGTC | TGAAGAATTTGCGCACCACTAC |
| 158 | mtrD-03 | CCGCCAAGCCGATATAGACA | GGCCGGGTTGCCAAA |
| 159 | ndm-1 | ATTAGCCGCTGCATTGAT | CATGTCGAGATAGGAAGTG |
| 160 | nimE | TGCGCCAAGATAGGGCATA | GTCGTGAATTCGGCAGGTTTA |
| 161 | nisB | GGGAGAGTTGCCGATGTTGTA | AGCCACTCGTTAAAGGGCAAT |
| 162 | oleC | CCCGGAGTCGATGTTCGA | GCCGAAGACGTACACGAACAG |
| 163 | oprD | ATGAAGTGGAGCGCCATTG | GGCCACGGCGAACTGA |
| 164 | oprJ | ACGAGAGTGGCGTCGACAA | AAGGCGATCTCGTTGAGGAA |
| 165 | pbp | CCGGTGCCATTGGTTTAGA | AAAATAGCCGCCCCAAGATT |
| 166 | pbp2x | TTTCATAAGTATCTGGACATGGAAGAA | CCAAAGGAAACTTGCTTGAGATTAG |
| 167 | Pbp5 | GGCGAACTTCTAATTAATCCTATCCA | CGCCGATGACATTCTTCTTATCTT |
| 168 | penA | AGACGGTAACGTATAACTTTTTGAAAGA | GCGTGTAGCCGGCAATG |
| 169 | pikR1 | TCGACATGCGTGACGAGATT | CCGCGAATTAGGCCAGAA |
| 170 | pikR2 | TCGTGGGCCAGGTGAAGA | TTCCCCTTGCCGGTGAA |
| 171 | pmrA | TTTGCAGGTTTTGTTCCTAATGC | GCAGAGCCTGATTTCTCCTTTG |
| 172 | pncA | GCAATCGAGGCGGTGTTC | TTGCCGCAGCCAATTCA |
| 173 | putitive multidrug | AATTTTGCCGATTATTGCTGAAA | GATTGTCATCATTCGTTTATCACCAA |
| 174 | qac | CAATAATAACCGAAATAATAGGGACAAGTT | AATAAGTGTTCCTAGTGTTGGCCATAG |
| 175 | qacA | TGGCAATAGGAGCTATGGTGTTT | AAGGTAACACTATTTTCGGTCCAAATC |
| 176 | qacA/qacB | TTTAGGCAGCCTCGCTTCA | CCGAATCCAAATAAAACCCAATAA |
| 177 | qacEdelta1-01 | TCGCAACATCCGCATTAAAA | ATGGATTTCAGAACCAGAGAAAGAAA |
| 178 | qacEdelta1-02 | CCCCTTCCGCCGTTGT | CGACCAGACTGCATAAGCAACA |
| 179 | qacH-01 | GTGGCAGCTATCGCTTGGAT | CCAACGAACGCCCACAA |
| 180 | qacH-02 | CATCGTGCTTGTGGCAGCTA | TGAACGCCCAGAAGTCTAGTTTT |
| 181 | qnrA | AGGATTTCTCACGCCAGGATT | CCGCTTTCAATGAAACTGCAA |
| 182 | rarD-02 | TGACGCATCGCGTGATCT | AAATTTTCTGTGGCGTCTGAATC |
| 183 | sat4 | GAATGGGCAAAGCATAAAAACTTG | CCGATTTTGAAACCACAATTATGATA |
| 184 | sdeB | CACTACCGCTTCCGCACTTAA | TGAAAAAACGGGAAAAGTCCAT |
| 185 | spcN-01 | AAAAGTTCGATGAAACACGCCTAT | TCCAGTGGTAGTCCCCGAATC |
| 186 | spcN-02 | CAGAATCTTCCTGAAAAGTTTGATGAA | CGCAGACACGCCGAATC |
| 187 | speA | GCAAGAGGTATTTGCTCAACAAGA | CAGGGTCACCCTCATAAAGAAAA |
| 188 | str | AATGAGTTTTGGAGTGTCTCAACGTA | AATCAAAACCCCTATTAAAGCCAAT |
| 189 | strA | CCGGTGGCATTTGAGAAAAA | GTGGCTCAACCTGCGAAAAG |
| 190 | strB | GCTCGGTCGTGAGAACAATCT | CAATTTCGGTCGCCTGGTAGT |
| 191 | sul1 | CAGCGCTATGCGCTCAAG | ATCCCGCTGCGCTGAGT |
| 192 | sul2 | TCATCTGCCAAACTCGTCGTTA | GTCAAAGAACGCCGCAATGT |
| 193 | sulA/folP-01 | CAGGCTCGTAAATTGATAGCAGAAG | CTTTCCTTGCGAATCGCTTT |
| 194 | sulA/folP-03 | CACGGCTTCGGCTCATGT | TGCCATCCTGTGACTAGCTACGT |
| 195 | tet(32) | CCATTACTTCGGACAACGGTAGA | CAATCTCTGTGAGGGCATTTAACA |
| 196 | tet(34) | CTTAGCGCAAACAGCAATCAGT | CGGTGATACAGCGCGTAAACT |
| 197 | tet(35) | ACCCCATGACGTACCTGTAGAGA | CAACCCACACTGGCTACCAGTT |
| 198 | tet(36)-01 | AGAATACTCAGCAGAGGTCAGTTCCT | TGGTAGGTCGATAACCCGAAAAT |
| 199 | tet(36)-02 | TGCAGGAAAGACCTCCATTACAG | CTTTGTCCACACTTCCACGTACTATG |
| 200 | tet(37) | GAGAACGTTGAAAAGGTGGTGAA | AACCAAGCCTGGATCAGTCTCA |
| 201 | tetA-01 | GCTGTTTGTTCTGCCGGAAA | GGTTAAGTTCCTTGAACGCAAACT |
| 202 | tetA-02 | CTCACCAGCCTGACCTCGAT | CACGTTGTTATAGAAGCCGCATAG |
| 203 | tetB-01 | AGTGCGCTTTGGATGCTGTA | AGCCCCAGTAGCTCCTGTGA |
| 204 | tetB-02 | GCCCAGTGCTGTTGTTGTCAT | TGAAAGCAAACGGCCTAAATACA |
| 205 | tetC-01 | CATATCGCAATACATGCGAAAAA | AAAGCCGCGGTAAATAGCAA |
| 206 | tetC-02 | ACTGGTAAGGTAAACGCCATTGTC | ATGCATAAACCAGCCATTGAGTAAG |
| 207 | tetD-01 | TGCCGCGTTTGATTACACA | CACCAGTGATCCCGGAGATAA |
| 208 | tetD-02 | TGTCATCGCGCTGGTGATT | CATCCGCTTCCGGGAGAT |
| 209 | tetE | TTGGCGCTGTATGCAATGAT | CGACGACCTATGCGATCTGA |
| 210 | tetG-01 | TCAACCATTGCCGATTCGA | TGGCCCGGCAATCATG |
| 211 | tetG-02 | CATCAGCGCCGGTCTTATG | CCCCATGTAGCCGAACCA |
| 212 | tetH | TTTGGGTCATCTTACCAGCATTAA | TTGCGCATTATCATCGACAGA |
| 213 | tetJ | GGGTGCCGCATTAGATTACCT | TCGTCCAATGTAGAGCATCCATA |
| 214 | tetK | CAGCAGTCATTGGAAAATTATCTGATTATA | CCTTGTACTAACCTACCAAAAATCAAAATA |
| 215 | tetL-01 | AGCCCGATTTATTCAAGGAATTG | CAAATGCTTTCCCCCTGTTCT |
| 216 | tetL-02 | ATGGTTGTAGTTGCGCGCTATAT | ATCGCTGGACCGACTCCTT |
| 217 | tetM-01 | CATCATAGACACGCCAGGACATAT | CGCCATCTTTTGCAGAAATCA |
| 218 | tetM-02 | TAATATTGGAGTTTTAGCTCATGTTGATG | CCTCTCTGACGTTCTAAAAGCGTATTAT |
| 219 | tetO-01 | ATGTGGATACTACAACGCATGAGATT | TGCCTCCACATGATATTTTTCCT |
| 220 | tetPA | AGTTGCAGATGTGTATAGTCGTAAACTATCTATT | TGCTACAAGTACGAAAACAAAACTAGAA |
| 221 | tetPB-01 | ACACCTGGACACGCTGATTTT | ACCGTCTAGAACGCGGAATG |
| 222 | tetPB-02 | TGATACACCTGGACACGCTGAT | CGTCCAAAACGCGGAATG |
| 223 | tetPB-03 | TGGGCGACAGTAGGCTTAGAA | TGACCCTACTGAAACATTAGAAATATACCT |
| 224 | tetPB-04 | AGTGGTGCAAATACTGAAAAAGTTGT | TTTGTTCCTTCGTTTTGGACAGA |
| 225 | tetPB-05 | CTGAAGTGGAGCGATCATTCC | CCCTCAACGGCAGAAATAACTAA |
| 226 | tetQ | CGCCTCAGAAGTAAGTTCATACACTAAG | TCGTTCATGCGGATATTATCAGAAT |
| 227 | tetR-02 | CGCGATAGACGCCTTCGA | TCCTGACAACGAGCCTCCTT |
| 228 | tetR-03 | CGCGATGGAGCAAAAGTACAT | AGTGAAAAACCTTGTTGGCATAAAA |
| 229 | tetS | TTAAGGACAAACTTTCTGACGACATC | TGTCTCCCATTGTTCTGGTTCA |
| 230 | tetT | CCATATAGAGGTTCCACCAAATCC | TGACCCTATTGGTAGTGGTTCTATTG |
| 231 | tetU-01 | GTGGCAAAGCAACGGATTG | TGCGGGCTTGCAAAACTATC |
| 232 | tetV | GCGGGAACGACGATGTATATC | CCGCTATCTCACGACCATGAT |
| 233 | tetX | AAATTTGTTACCGACACGGAAGTT | CATAGCTGAAAAAATCCAGGACAGTT |
| 234 | tnpA-01 | CATCATCGGACGGACAGAATT | GTCGGAGATGTGGGTGTAGAAAGT |
| 235 | tnpA-02 | GGGCGGGTCGATTGAAA | GTGGGCGGGATCTGCTT |
| 236 | tnpA-03 | AATTGATGCGGACGGCTTAA | TCACCAAACTGTTTATGGAGTCGTT |
| 237 | tnpA-04 | CCGATCACGGAAAGCTCAAG | GGCTCGCATGACTTCGAATC |
| 238 | tnpA-05 | GCCGCACTGTCGATTTTTATC | GCGGGATCTGCCACTTCTT |
| 239 | tnpA-07 | GAAACCGATGCTACAATATCCAATTT | CAGCACCGTTTGCAGTGTAAG |
| 240 | tolC-01 | GGCCGAGAACCTGATGCA | AGACTTACGCAATTCCGGGTTA |
| 241 | tolC-02 | CAGGCAGAGAACCTGATGCA | CGCAATTCCGGGTTGCT |
| 242 | tolC-03 | GCCAGGCAGAGAACCTGATG | CGCAATTCCGGGTTGCT |
| 243 | Tp614 | GGAAATCAACGGCATCCAGTT | CATCCATGCGCTTTTGTCTCT |
| 244 | ttgA | ACGCCAATGCCAAACGATT | GTCACGGCGCAGCTTGA |
| 245 | ttgB | TCGCCCTGGATGTACACCTT | ACCATTGCCGACATCAACAAC |
| 246 | vanA | AAAAGGCTCTGAAAACGCAGTTAT | CGGCCGTTATCTTGTAAAAACAT |
| 247 | vanB-01 | TTGTCGGCGAAGTGGATCA | AGCCTTTTTCCGGCTCGTT |
| 248 | vanB-02 | CCGGTCGAGGAACGAAATC | TCCTCCTGCAAAAAAAGATCAAC |
| 249 | vanC-01 | ACAGGGATTGGCTATGAACCAT | TGACTGGCGATGATTTGACTATG |
| 250 | vanC-03 | AAATCAATACTATGCCGGGCTTT | CCGACCGCTGCCATCA |
| 251 | vanC1 | AGGCGATAGCGGGTATTGAA | CAATCGTCAATTGCTCATTTCC |
| 252 | vanC2/vanC3 | TTTGACTGTCGGTGCTTGTGA | TCAATCGTTTCAGGCAATGG |
| 253 | vanG | ATTTGAATTGGCAGGTATACAGGTTA | TGATTTGTCTTTGTCCATACATAATGC |
| 254 | vanHB | GAGGTTTCCGAGGCGACAA | CTCTCGGCGGCAGTCGTAT |
| 255 | vanHD | GTGGCCGATTATACCGTCATG | CGCAGGTCATTCAGGCAAT |
| 256 | vanRA-01 | CCCTTACTCCCACCGAGTTTT | TTCGTCGCCCCATATCTCAT |
| 257 | vanRA-02 | CCACTCCGGCCTTGTCATT | GCTAACCACATTCCCCTTGTTTT |
| 258 | vanRB | GCCCTGTCGGATGACGAA | TTACATAGTCGTCTGCCTCTGCAT |
| 259 | vanRC | TGCGGGAAAAACTGAACGA | CCCCCCATACGGTTTTGATTA |
| 260 | vanRC4 | AGTGCTTTGGCTTATCTCGAAAA | TCCGGCAGCATCACATCTAA |
| 261 | vanRD | TTATAATGGCAAGGATGCACTAAAGT | CGTCTACATCCGGAAGCATGA |
| 262 | vanSA | CGCGTCATGCTTTCAAAATTC | TCCGCAGAAAGCTCAATTTGTT |
| 263 | vanSB | GCGCGGCAAATGACAAC | TTTGCCATTTTATTCGCACTGT |
| 264 | vanSC-02 | GCCATCAGCGAGTCTGATGA | CAGCTGGGATCGTTTTTCCTT |
| 265 | vanSE | TGGCCGAAGAAGCAGGAA | CAATAATACTCGTCAAAGGAGTTCTCA |
| 266 | vanTC-01 | CACACGCATTTTTTCCCATCTAG | CAGCCAACAGATCATCAAAACAA |
| 267 | vanTC-02 | ACAGTTGCCGCTGGTGAAG | CGTGGCTGGTCGATCAAAA |
| 268 | vanTE | GTGGTGCCAAGGAAGTTGCT | CGTAGCCACCGCAAAAAAAT |
| 269 | vanTG | CGTGTAGCCGTTCCGTTCTT | CGGCATTACAGGTATATCTGGAAA |
| 270 | vanWB | CGGACAAAGATACCCCCTATAAAG | AAATAGTAAATTGCTCATCTGGCACAT |
| 271 | vanWG | ACATTTTCATTTTGGCAGCTTGTAC | CCGCCATAAGAGCCTACAATCT |
| 272 | vanXA | CGCTAAATATGCCACTTGGGATA | TCAAAAGCGATTCAGCCAACT |
| 273 | vanXB | AGGCACAAAATCGAAGATGCTT | GGGTATGGCTCATCAATCAACTT |
| 274 | vanXD | TAAACCGTGTTATGGGAACGAA | GCGATAGCCGTCCCATAAGA |
| 275 | vanYB | GGCTAAAGCGGAAGCAGAAA | GATATCCACAGCAAGACCAAGCT |
| 276 | vanYD-01 | AAGGCGATACCCTGACTGTCA | ATTGCCGGACGGAAGCA |
| 277 | vanYD-02 | CAAACGGAAGAGAGGTCACTTACA | CGGACGGTAATAGGGACTGTTC |
| 278 | vatB-01 | GGAAAAAGCAACTCCATCTCTTGA | TCCTGGCATAACAGTAACATTCTGA |
| 279 | vatB-02 | TTGGGAAAAAGCAACTCCATCT | CAATCCACACATCATTTCCAACA |
| 280 | vatC-01 | CGGAAATTGGGAACGATGTT | GCAATAATAGCCCCGTTTCCTA |
| 281 | vatC-02 | CGATGTTTGGATTGGACGAGAT | GCTGCAATAATAGCCCCGTTT |
| 282 | vatE-01 | GGTGCCATTATCGGAGCAAAT | TTGGATTGCCACCGACAAT |
| 283 | vatE-02 | GACCGTCCTACCAGGCGTAA | TTGGATTGCCACCGACAATT |
| 284 | vgaA-01 | CGAGTATTGTGGAAAGCAGCTAGTT | CCCGTACCGTTAGAGCCGATA |
| 285 | vgaA-02 | GACGGGTATTGTGGAAAGCAA | TTTCCTGTACCATTAGATCCGATAATT |
| 286 | vgb-01 | AGGGAGGGTATCCATGCAGAT | ACCAAATGCGCCCGTTT |
| 287 | vgbB-01 | CAGCCGGATTCTGGTCCTT | TACGATCTCCATTCAATTGGGTAAA |
| 288 | vgbB-02 | ATACGAGCTGCCTAATAAAGGATCTT | TGTGAACCACAGGGCATTATCA |
| 289 | yceE/mdtG-01 | TGGCACAAAATATCTGGCAGTT | TTGTGTGGCGATAAGAGCATTAG |
| 290 | yceE/mdtG-02 | TTATCTGTTTTCTGCTCACCTTCTTTT | GCGTGGTGACAAACAGGCTTA |
| 291 | yceL/mdtH-01 | TCGGGATGGTGGGCAAT | CGATAACCGAGCCGATGTAGA |
| 292 | yceL/mdtH-02 | CGCGTGAAACCTTAAGTGCTT | AGACGGCTAAACCCCATATAGCT |
| 293 | yceL/mdtH-03 | CTGCCGTTAAATGGATGTATGC | ACTCCAGCGGGCGATAGG |
| 294 | yidY/mdtL-01 | GCAGTTGCATATCGCCTTCTC | CTTCCCGGCAAACAGCAT |
| 295 | 16S rRNA | 338F(ACTCCTACGGGAGGCAGCA) | 806R(GGACTACHVGGGTWTCTAAT) |
