# Supplementary material for: The proliferation of antibiotic resistance genes (ARGs) and microbial communities in industrial wastewater treatment plant treating N,N-dimethylformamide (DMF) by AAO process
Source: PLoS One. 2024 Apr 10;19(4):e0299740. doi: 10.1371/journal.pone.0299740 (PMC11006197; doi:10.1371/journal.pone.0299740)
Supplement: Attachment — Submitted filename: PONE-D-23-27587(1).docx [file pone.0299740.s003.docx]

Review Comments: PONE-D-23-27587

In this study, wastewater from five stages of the AAO process at a designated water plant was collected and analyzed for COD, DMF, ARGs, and microbial community structure. Results showed that the AAO process may act as a microbial source that increases the total abundance of ARGs, and the wastewater had higher abundance of ARGs for non-pathogenic bacteria than for pathogenic bacteria. Overall, this study provides information on microbial community structure and the mechanisms driving changes in the abundance of ARGs in industrial wastewater treatment plants. However, before considering publication, the following questions should be addressed. My specific comments are as follows:

1. Authors should be aware of spelling and formatting errors, such as line 67.
2. This study sampled only one specific water plant which is not representative and the conclusions may not be generalizable. The authors should add some related references such as Journal of Environmental Management,Volume 347, 2023, 119053, Journal of Hazardous Materials,Volume 298, 2015, Pages 303-309, Process Safety and Environmental Protection,Volume 93, 2015, Pages 68-74.
3. COD was consistent with the amount of DMF. Is there a way to add a correlation chart and embellish Figure 1？
4. Abbreviations need to be spelled out in full the first time they appear, DMF,AAO in the abstract, PCA in the methodology on line 31;MGEs in the results；in abbreviations should be placed after the corresponding full name, not at the end of each sentence.
5. In the conclusions, it is stated that the DMFs in Figure 2b for AP, ANP, AEP, but the graph shows significantly higher values for AP than for influent and effluent.Is this due to the lack of graphs to allow for the corresponding conclusions to be drawn?
