# Supplementary material for: The proliferation of antibiotic resistance genes (ARGs) and microbial communities in industrial wastewater treatment plant treating N,N-dimethylformamide (DMF) by AAO process
Source: PLoS One. 2024 Apr 10;19(4):e0299740. doi: 10.1371/journal.pone.0299740 (PMC11006197; doi:10.1371/journal.pone.0299740)
Supplement: Attachment — Submitted filename: Response to Reviewers.docx [file pone.0299740.s004.docx]

Thank you for taking the time to review our manuscript. We appreciate your constructive feedback, and we have carefully considered your comments. Below are our responses addressing the specific concerns you raised:

Reviewer #1：

1．This work uses a very powerful tool to analyze all the resistome in environment samples. The rationale to support analyzing a WWPT degrading the DMF is weak, and the discussion of the results is superficial. The methodology is very general, and many details are missing.

**Reply:** Thank you for your valuable feedback. We have taken your suggestions into consideration and have made some enhancements to our submission. Specifically, we have added more details regarding the methods employed in our study as follow:

“Determination and analysis of DMF content

The analysis was conducted using gas chromatography-mass spectrometry, a highly reliable and precise method. In order to test for the presence of DMF in the sample, it was first transferred to a suitable organic solvent and dissolved to create a DMF solution with a specific concentration. To ensure accurate results, a series of standard solutions with varying concentrations of DMF were prepared. These standard solutions covered a range of concentrations that could potentially be found in the sample under investigation. Both the sample and the standard solutions were then injected into the gas chromatograph. Careful consideration was given to the selection of the gas chromatograph column and mobile phase, ensuring optimal conditions for analysis. For qualitative identification, the mass spectrum of the DMF in the sample was compared to that of a known DMF reference. This comparison allowed for the confident identification of DMF in the sample. Quantitative analysis was carried out by comparing the peak area of DMF in the sample to that of the standard curve. By establishing the relationship between the peak area and the concentration at each point on the standard curve, a linear regression analysis was performed. This analysis enabled the accurate calculation of the DMF concentration in the sample under investigation.”

In the discussion, we provided additional clarification on the relationship between microbial communities, antibiotic resistance genes (ARGs), and dissolved organic matter (DMF).

“Previous studies [1] have extensively investigated a total of 20,762 metagenomes, revealing the presence of DMF-degrading bacteria in 952 genomes. These bacteria, predominantly belonging to the Rhizobiales, Paracoccus, Rhodococcus, Achromobacter, and Pseudomonas genera, are primarily aerobic and are found within the Proteobacteria and Actinobacteria phyla. Intriguingly, these two phyla are also known to be enriched with antibiotic resistance genes (ARGs). Based on these findings, we propose a hypothesis that suggests that these bacteria play a crucial role in the degradation of DMF during sewage treatment. However, an unintended consequence of their presence may be the increased enrichment of ARGs, thereby elevating the abundance of these genes in the treated wastewater. While this assumption is currently based solely on experimental results, it presents a compelling argument suggesting that the resistant bacteria community may facilitate the reduction of DMF levels in sewage.”

2．Define AAO acronym in the abstract

**Reply**: Thanks for your advice. Adjustments have been made to follow your suggestions, and abbreviations are spelled out in full when they first appear.

3．May the Authors support the statements about introduced bacteria? “it is common to introduce these bacteria into WWPTs to degrade the DMF.” Or “However, the introduced bacteria may be the major driver impacting the ARGs profile. Although the biological treatment could reduce DMF significantly, the abundance of ARGs in the effluents may be high. and effluents may favor the persistence and spread of antibiotic resistance in the microbial communities of the receiving environments.”

**Reply**：Thank you for your thoughtful consideration of our manuscript. We acknowledge the importance of providing adequate support for statements regarding the introduction of bacteria into wastewater treatment plants (WWPTs) for DMF degradation. In the revised manuscript, we will include additional references and data to substantiate the claim that it is common practice to introduce specific bacteria for the purpose of DMF degradation. This will enhance the credibility and robustness of the statements in question. “it is common to introduce these bacteria into WWPTs to degrade the DMF [2].” “However, the introduced bacteria may be the major driver impacting the ARGs profile. Although the biological treatment could reduce DMF significantly, the abundance of ARGs in the effluents may be high. and effluents may favor the persistence and spread of antibiotic resistance in the microbial communities of the receiving environments,” A recent study has shown that effluents from wastewater treatment plants are a significant source of antibiotic-resistant bacteria and antibiotic-resistant genes (ARGs), posing a significant risk to public health. Therefore, We propose a hypothesis that the treatment of DMF, by introducing degradation bacteria, may lead to the dissemination and spread of antibiotic resistance genes (ARGs).

4．The introduction may be more supportive of the relevance of analyzing ARGs in a DMF containing wastewater. Why could this chemical be relevant to ARG or their transferring in the microbial community?

**Reply:** Thank you for your thoughtful consideration of our manuscript. The existence of ARGs in microorganisms may be related to environmental stress, such as metal can promote microbial strains to produce resistance, which is related to the co-evolution of genes. As a commonly used chemical raw material, DMF may promote the existence or transfer of some ARGs in strains through this co-evolution in the environment.

5．The introduction and final paragraph seem to be a summary of the methodology and results. In the authors’ guidelines, only a conclusive brief statement is required. Please be more specific.

**Reply:** Thank you for your thoughtful consideration of our manuscript. The summary description of the last paragraph of the introduction was given. “This study aimed to investigate the effects of DMF on the distribution of ARGs during the AAO process in industrial WWTPs. It also examined changes in bacterial community structures and ARGs occurrence during the process, as well as the relationships between ARGs and bacterial genera, specifically pathogens. The results showed that DMF content decreased during the AAO process, while the abundance and number of ARGs and bacterial hosts increased. The study also found that the potential transfer of ARGs in pathogenic bacteria decreased after the AAO process. However, some ARGs still pose a significant threat to public health through their distribution and accumulation in non-pathogenic bacteria, which can be discharged through effluent. Further measures may be necessary to address this issue.”

6．Could the authors may be specific about their “samples replicates”?. Do sampling was carried out on different days? Please specify

**Reply:** Thank you for your thoughtful consideration of our manuscript. Sample duplication refers to three replicates taken at different locations at the same time. The original text "For each sample there were three independent replicates." has been changed to "Repeat sampling of each sample three times at different locations at the same time."

7．Line 106. Please justify “The abundance of expressed ARGs”, if the analysis was DNA-based, authors can’t claim “gene expression”. In the same sense, lines 120, 129 mention transcripts. Is it correct?

**Reply:** Thank you for your advice, and I will revise it. Delete inappropriate statements, such as”expressed” ”transcripts”.

8．The methodology for DMF analysis is missing.

**Reply:** Thank you for your advice. We provide the following supplementary explanations: “The analysis was conducted using gas chromatography-mass spectrometry, a highly reliable and precise method. In order to test for the presence of DMF in the sample, it was first transferred to a suitable organic solvent and dissolved to create a DMF solution with a specific concentration. To ensure accurate results, a series of standard solutions with varying concentrations of DMF were prepared. These standard solutions covered a range of concentrations that could potentially be found in the sample under investigation. Both the sample and the standard solutions were then injected into the gas chromatograph. Careful consideration was given to the selection of the gas chromatograph column and mobile phase, ensuring optimal conditions for analysis. For qualitative identification, the mass spectrum of the DMF in the sample was compared to that of a known DMF reference. This comparison allowed for the confident identification of DMF in the sample. Quantitative analysis was carried out by comparing the peak area of DMF in the sample to that of the standard curve. By establishing the relationship between the peak area and the concentration at each point on the standard curve, a linear regression analysis was performed. This analysis enabled the accurate calculation of the DMF concentration in the sample under investigation.”

9．Table S1 is missing.

**Reply:** Thanks for your advice. Table S1 has been replenished.

10．How the 16S rRNA Absolute gene abundance was quantified? This methodology is missing. How do the authors explain a higher abundance in the effluent (Fig 1c)? The authors may explain better the process in the WWTP and their conditions? Does the WWTP have a settling unit for aerobic flocs? The units of 16S rRNA concentration in Figure 1c are missing.

**Reply:** Thank you for your thoughtful consideration of our manuscript. We provide the following supplementary detail: “While the active bacterial community was profiled by amplicon sequencing the 16S rRNA gene. To assess the diversity and relative abundance of bacteria in all samples, the V3 and V4 region of 16S rRNA was amplified .The initial enzyme activation was performed at 95 °C for 5 min, and then 35 cycles of the following program were used for amplification: 95 °C for 30 s, 58 °C for 30 s and 72 °C for 30 s. By sequenced with Illumina Hiseq 2500 platform universal primers 341F and 806R by Yuanzai biotechnology Co., Ltd (Hefei, China). The raw pair-end reads were assembled after filtering of adaptor, low-quality reads, ambiguous nucleotides, and barcodes to generate clean joined reads capturing the complete V4-V5 region of the 16S rRNA gene. Quantitative Insights Into Microbial Ecology (QIIME) was used for further data processing [3].”

Sewage treatment plants have aerobic flocculation sedimentation units, which use aerobic bacteria and flocculants to remove organic matter and suspended matter in sewage. The aerobic flocculation sedimentation device is generally used to treat high-concentration industrial wastewater. The aerobic flocculation sedimentation device can improve the abundance of flora, because the aerobic bacteria can use the organic matter in the sewage as a carbon source and energy source to grow and reproduce. At the same time, the flocculant can increase the flocculation of the flora, making it easier for the flora to precipitate and return, so as to maintain the high concentration of flora. The increased abundance of 16S rRNA at the water outlet may be caused by the decreased flocculation effect. 16S rRNA concentration units have been added in Fig 1D

11．Line 157. Why the different IN clustering was so “obviously”?

**Reply:** Thank you for your thoughtful consideration of our manuscript. The original text was “In addition, the replicated samples of ANP, AEP, and EF samples were clustered while that of IN is obviously separated which indicated that the ARGs abundances were more stable in treatments than in IN.” IN is not only visibly dispersed but also exhibits a clear lack of clustering due to the diverse sources of sewage within the area. However, it is important to note that after undergoing the unified AAO treatment, a remarkable clustering phenomenon is expected to occur. The initial observation of IN's dispersion can be attributed to the varied origins of sewage within the region. Different industrial, residential, and commercial sources contribute to a wide distribution of pollutants, making it difficult to identify specific clusters. This dispersion is evident in the inconsistent patterns and irregularities observed in the sewage samples.

12．Define the MGE acronym.

**Reply:** Thanks for your advice. Modifications have been made in accordance with your suggestions.

13．The authors may support this discussion with a deep literature review: “We infer that the increase abundance of ARGs in WWTPs may be related to bacterium community, because many previous studies have demonstrated that some phylum of strain was closely related to the enrichment of ARGs.”

**Reply:** Thank you for your thoughtful consideration of our manuscript. The literature supports this conclusion.“Infer that the increase abundance of ARGs in WWTPs may be related to bacterium community, because many previous studies have demonstrated that some phylum of strain was closely related to the enrichment of ARGs [4, 5].”

14．Figure 2E, is lacking the label corresponding to each pie chart.

**Reply:** Thanks for your advice. The corresponding label has been added

15．How did the authors select the pathogenic bacteria from the whole bacteria community? This methodology is unclear.

**Reply:** Thank you for your thoughtful consideration of our manuscript. The pathogenic bacteria were selected based on solutions mentioned in previous literature [6].

16．The authors may justify this statement “Specifically, DMF had a significant positive correlation with Bacillus, which was belonging to Firmicutes, indicated that DMF may promote the growth of Bacillus. Furthermore”. A positive correlation doesn’t mean a biological activity. This is the only discussion about the effect of DMF.. So, it seems irrelevant to the study, which contradicts the justification in the introduction. Authors may reconsider if DMF can be a determinant parameter to study.

**Reply:** Thank you for your thoughtful consideration of our manuscript. The positive correlation between DMF and bacillus does not directly indicate that DMF may promote the growth of Bacillus. What I want to show is that DMF may make Bacillus a competitive advantage by inhibiting the growth of other strains. Amend the original to“Specifically, DMF had a significant positive correlation with Bacillus, which was belonging to Firmicutes, indicated that DMF may make Bacillus a competitive advantage by inhibiting the growth of other strains.”

Reviewer #2:

1 First person must not be used in the manuscript, such as our, we.

**Reply:** Thanks for your advice. First person has been modified.

2 In Abstract, the DMF should be first given the complete name, not only abbreviation.

**Reply:** Thanks for your advice. Adjustments have been made to follow your suggestions, and abbreviations are spelled out in full when they first appear.

3 the keywords were not approviate, DMF should be included.

**Reply:** Thanks for your advice. Keywords have been added,such as DMF.

4 The instrument of HT-qPCR should be given.

**Reply:** Thank you for your thoughtful consideration of our manuscript. The original text "The abundance of expressed ARGs were characterize by high throughput quantitative PCR on the Real time PCR." has been changed to "The abundance of expressed ARGs were characterize by high throughput quantitative PCR (HT-qPCR). HT-qPCR was performed using the Wafergen SmartChip Real-time PCR system (Wafergen, Fremont, CA). This SmartChip platform can be used for large-scale analysis by processing 5184-nanowell reactions per run."

5 The gene name should be italic.

**Reply**: Gene names are italicized, thanks for your advice.

6 The ARGs should be analyzed by different category.

**Reply:** Thank you for your thoughtful consideration of our manuscript. By analyzing 11 different ARGs, we analyzed the variation of ARGs type and abundance during AAO treatment. Sewage treatment did not change the composition of the main types of ARGs (Fig 2B-D). However, the quantity changed during treatment, for example, the inlet was mainly composed of aminoglycoside, multidrug and transposase, while the outlet was mainly composed of MGEs, multidrug and transposase (Fig 2C). In AP, ANP and AEP, multidrug, transposase and sulfanilamide are the main ARGs(Fig 2D). MexF, tna-04, and qacEdelta1-02 are specific ARGs.

7 The component of wastewater have an effect on abundance of ARGs this sentence should be changed, for “have an effect” was nonsense.

**Reply:** The original text "The component of wastewater have an effect on abundance of ARGs" has been changed to "The correlation between ARGs and the composition of wastewater"

8 The effect of DMF should be focused.

**Reply:** Thank you for your advice. we focused the effect of DMF. “DMF, a component of industrial wastewater, poses a significant threat to both aquatic organisms and the environment. Its discharge into water bodies can lead to detrimental effects on aquatic communities[7], altering their ecological structure and function. Additionally, DMF has the potential to inhibit the growth and activity of soil microorganisms, ultimately disrupting the stability and functionality of soil ecosystems [8]. Furthermore, it is important to highlight the adverse impact of DMF on human health. Prolonged exposure to high concentrations of DMF can result in various health issues, including skin and respiratory irritation, as well as liver and kidney damage [9]. These potential health risks necessitate the urgent need for effective DMF removal methods. Conventional biological processing methods have proven to be ineffective in removing DMF due to its high solubility and stability. As a result, it becomes crucial to explore alternative treatment processes that can efficiently eliminate DMF from wastewater. In this regard, the AAO treatment process has emerged as a promising solution. With its capability to effectively remove DMF, the AAO treatment process offers a viable method for addressing the presence of DMF in wastewater. By employing this innovative approach, we can mitigate the negative impacts of DMF on wastewater treatment plants and ensure their optimal functioning. “

9 The effect of MGEs should be focused.

**Reply:** Thank you for your advice. we focused the effect of MGEs. “MGEs are fragments of DNA that can move around the genome, including transposons, integrons, and plasmids. They are capable of horizontal gene transfer between the genetic material of the bacteria, thereby rapidly spreading antibiotic resistance genes through the bacterial population [10]. The spread of MGEs poses a challenge to antibiotic treatment. Because they enable bacteria to quickly acquire antibiotic resistance genes, which can cause antibiotics to become ineffective [11]. In addition, because MGEs are highly plastic and adaptable, they can also promote the evolution and diversification of antibiotic resistance genes [12]. This means that antibiotic use can select not only antibiotic-resistant bacteria, but also bacteria that carry MGEs, further exacerbating the antibiotic resistance problem. Therefore, understanding the effect of MGEs on antibiotic resistance genes is of great significance for formulating rational antibiotic use strategies and antibiotic resistance prevention and control.”

Reviewer #3:
1. Authors should be aware of spelling and formatting errors, such as line 67.

**Reply:** Thank you for your suggestion. The adjustment has been made as per your recommendation. The original text "the abundance of ARGs in the effluents maybe high. and effluents may favor the persistence and spread of antibiotic resistance in the microbial communities of the receiving environments." has been changed to "the abundance of ARGs in the effluents maybe high. Effluents may favor the persistence and spread of antibiotic resistance in the microbial communities of the receiving environments." accordingly.

2. This study sampled only one specific water plant which is not representative and the conclusions may not be generalizable. The authors should add some related references for comparison such as Journal of Environmental Management,Volume 347, 2023, 119053, Journal of Hazardous Materials,Volume 298, 2015, Pages 303-309, Process Safety and Environmental Protection,Volume 93, 2015, Pages 68-74.

**Reply:** Thanks for your advice. References are used to support my conclusion [13-15].

3. COD was consistent with the amount of DMF. Is there a way to add a correlation chart and embellish Figure 1？

**Reply:** Thanks for your advice. We added the correlation figure Fig1C as suggested

4. Abbreviations need to be spelled out in full the first time they appear, DMF,AAO in the abstract, PCA in the methodology on line 31.MGEs in the result.in abbreviations should be placed after the corresponding full name, not at the end of each sentence.

**Reply:** Thanks for your advice. Adjustments have been made to follow your suggestions, and abbreviations are spelled out in full when they first appear

5. In the conclusions, it is stated that the DMFs in Figure 2b for AP, ANP, AEP, but the graph shows significantly higher values for AP than for influent and effluent.Is this due to the lack of graphs to allow for the corresponding conclusions to be drawn?

**Reply:** Thanks for your advice. Fig 1A shows the change of DMF content, during the treatment process, DMF content decreased significantly, with the highest IN and the lowest EF. Fig 2B is the relative copy number of ARGs and MGEs, with AP being the highest. Fig 2C is the Absolute copy number of ARGs and MGEs, the absolute abundance of ARGs and MGEs is increased. The expression in lines 159-162 is changed to ”Differentially relative abundant ARGs found in wastewater during the AAO process are displayed in Fig 2B, with AP being the highest. The application of activated sludge in AP, ANP, and AEP pool reduced the content of DMF(Fig 1A), however, it also significantly increased the absolute abundance of ARGs as well as MGEs leading to a great enrichment than IN (Fig 2C).”

1.	Li J, Dijkstra P, Lu Q, Wang S, Chen S, Li D, et al. Genomics-informed insights into microbial degradation of N, N-dimethylformamide. International Biodeterioration & Biodegradation. 2021;163:105283.

2.	Lu X, Wang W, Zhang L, Hu H, Xu P, Wei T, et al. Molecular Mechanism of N,N-Dimethylformamide Degradation in Methylobacterium sp. Strain DM1. Applied and environmental microbiology. 2019;85(12). Epub 2019/04/07. doi: 10.1128/aem.00275-19. PubMed PMID: 30952664; PubMed Central PMCID: PMCPMC6544821.

3.	Caporaso JG, Kuczynski J, Stombaugh J, Bittinger K, Bushman FD, Costello EK, et al. QIIME allows analysis of high-throughput community sequencing data. Nature methods. 2010;7(5):335-6. Epub 2010/04/13. doi: 10.1038/nmeth.f.303. PubMed PMID: 20383131; PubMed Central PMCID: PMCPMC3156573.

4.	Ju F, Zhang T. Bacterial assembly and temporal dynamics in activated sludge of a full-scale municipal wastewater treatment plant. The ISME journal. 2015;9(3):683-95. Epub 2014/09/03. doi: 10.1038/ismej.2014.162. PubMed PMID: 25180966; PubMed Central PMCID: PMCPMC4331583.

5.	Jia S, Shi P, Hu Q, Li B, Zhang T, Zhang XX. Bacterial Community Shift Drives Antibiotic Resistance Promotion during Drinking Water Chlorination. Environmental science & technology. 2015;49(20):12271-9. Epub 2015/09/24. doi: 10.1021/acs.est.5b03521. PubMed PMID: 26397118.

6.	Yu Q, Feng T, Yang J, Su W, Zhou R, Wang Y, et al. Seasonal distribution of antibiotic resistance genes in the Yellow River water and tap water, and their potential transmission from water to human. Environmental pollution (Barking, Essex : 1987). 2022;292(Pt A):118304. Epub 2021/10/11. doi: 10.1016/j.envpol.2021.118304. PubMed PMID: 34627965.

7.	Di Mauro V, Kamyab E, Kellermann MY, Moeller M, Nietzer S, Luetjens LH, et al. Ecotoxicological Effects of Four Commonly Used Organic Solvents on the Scleractinian Coral Montipora digitata. Toxics. 2023;11(4). Epub 2023/04/28. doi: 10.3390/toxics11040367. PubMed PMID: 37112595; PubMed Central PMCID: PMCPMC10146860.

8.	Ul'yanovskii NV, Lakhmanov DE, Pikovskoi, II, Falev DI, Popov MS, Kozhevnikov AY, et al. Migration and transformation of 1,1-dimethylhydrazine in peat bog soil of rocket stage fall site in Russian North. The Science of the total environment. 2020;726:138483. Epub 2020/04/22. doi: 10.1016/j.scitotenv.2020.138483. PubMed PMID: 32315849.

9.	Zhang Y, Pei M, Zhang B, He Y, Zhong Y. Changes of antibiotic resistance genes and bacterial communities in the advanced biological wastewater treatment system under low selective pressure of tetracycline. Water research. 2021;207:117834. Epub 2021/11/12. doi: 10.1016/j.watres.2021.117834. PubMed PMID: 34763281.

10.	Wang H, Hou L, Liu Y, Liu K, Zhang L, Huang F, et al. Horizontal and vertical gene transfer drive sediment antibiotic resistome in an urban lagoon system. Journal of environmental sciences (China). 2021;102:11-23. Epub 2021/02/28. doi: 10.1016/j.jes.2020.09.004. PubMed PMID: 33637236.

11.	Nguyen AQ, Vu HP, Nguyen LN, Wang Q, Djordjevic SP, Donner E, et al. Monitoring antibiotic resistance genes in wastewater treatment: Current strategies and future challenges. The Science of the total environment. 2021;783:146964. Epub 2021/04/19. doi: 10.1016/j.scitotenv.2021.146964. PubMed PMID: 33866168.

12.	Vasco K, Guevara N, Mosquera J, Zapata S, Zhang L. Characterization of the gut microbiome and resistome of Galapagos marine iguanas (Amblyrhynchus cristatus) from uninhabited islands. Animal microbiome. 2022;4(1):65. Epub 2022/12/15. doi: 10.1186/s42523-022-00218-4. PubMed PMID: 36517909; PubMed Central PMCID: PMCPMC9749353.

13.	Huang M-H, Zhang W, Liu C, Hu H-Y. Fate of trace tetracycline with resistant bacteria and resistance genes in an improved AAO wastewater treatment plant. Process Safety and Environmental Protection. 2015;93:68-74. doi: 10.1016/j.psep.2014.04.004.

14.	Czatzkowska M, Rolbiecki D, Zaborowska M, Bernat K, Korzeniewska E, Harnisz M. The influence of combined treatment of municipal wastewater and landfill leachate on the spread of antibiotic resistance in the environment - A preliminary case study. Journal of environmental management. 2023;347:119053. Epub 2023/09/26. doi: 10.1016/j.jenvman.2023.119053. PubMed PMID: 37748295.

15.	Huang M, Qi F, Wang J, Xu Q, Lin L. Changes of bacterial diversity and tetracycline resistance in sludge from AAO systems upon exposure to tetracycline pressure. Journal of hazardous materials. 2015;298:303-9. Epub 2015/06/17. doi: 10.1016/j.jhazmat.2015.05.039. PubMed PMID: 26079369.
