# Supplementary material for: The proliferation of antibiotic resistance genes (ARGs) and microbial communities in industrial wastewater treatment plant treating N,N-dimethylformamide (DMF) by AAO process
Source: PLoS One. 2024 Apr 10;19(4):e0299740. doi: 10.1371/journal.pone.0299740 (PMC11006197; doi:10.1371/journal.pone.0299740)
Supplement: Attachment — Submitted filename: Response to Reviewers0208.docx [file pone.0299740.s005.docx]

Dear Reviewer,

Thank you for taking the time to provide such a thorough and constructive review of our manuscript. We appreciate your positive comments about the comprehensive and detailed results of our study and are pleased that you found our research to be significant and timely. We also acknowledge your suggestions for improvement and have made the necessary revisions as per your comments.

1. Providing a graphical abstract can aid readers in better understanding the article.

**Reply**: Thank you for your advice. We have included a graphical abstract to help readers better understand the article's content. In line 112, we add the sentence“The experimental analysis process is shown in the graphical abstract (fig. S1).”

2. The current text seems to include conclusions within the results section. Typically, it is best to keep these sections separate, with the results section strictly presenting the findings, and interpretation or implications discussed in the conclusion section.

**Reply:** Thank you for your advice. We agree with your suggestion and have now separated the results and conclusion sections. The conclusions have been moved to a dedicated section where we discuss the interpretation and implications of our findings. We move the sentence “AAO process can remove the DMF efficiently and bacteria plays an important role in this process.” from result to conclusion.

3. There are several typos or grammatical errors that need to be corrected.

**Reply:** We apologize for any confusion that may have caused. We check the article again. All typos and grammatical errors have been corrected.

4. The writing style is quite passive. In scientific writing, active voice is often preferred as it is more direct and concise.

**Reply:** Thank you for your advice. We have revised the manuscript to incorporate a more active voice, making it more direct and concise.

5. Line 106, "The wastewater was collected from the Shen Nuobei industrial wastewater treatment plant (118.507° N, 31.689° E)，Ma’anshan city, Anhui province, China, in December 2021.", suggest to change to "In December 2021, wastewater was sampled from the Shen Nuobei industrial wastewater treatment plant located in Ma'anshan city, Anhui province, China (118.507° N, 31.689° E)."

**Reply:** Thank you for your advice. The sentence on Line 106 has been revised as suggested.

6. Line 110, "Collected 2 L of wastewater samples from influent, anaerobic, anoxic, aerobic, and effluent tanks.", suggest to change to "Two liters of wastewater samples were collected from the influent, anaerobic, anoxic, aerobic, and effluent tanks."

**Reply:** Thank you for your advice. The sentence on Line 110 has been revised as per your suggestion.

7. Line 111, "Repeat sampling of each sample three times at different locations at the same time.", suggest to change to "Each sample was collected three times from different locations concurrently."

**Reply:** Thank you for your advice. The sentence on Line 111 has been revised to clarify that each sample was collected three times from different locations concurrently.

8. Line 301, "ARGs has increased rapidly" should be changed to "ARGs have increased rapidly".

**Reply:** Thank you for your advice. The sentence on Line 301 has been corrected to "ARGs have increased rapidly".

9. Line 303, "a previous research" should be changed to "previous research".

**Reply:** Thank you for your advice. The phrase "a previous research" on Line 303 has been corrected to "previous research".

10. Line 305, "lead to a significant increase" should be changed to "leading to a significant increase".

**Reply:** Thank you for your advice. The phrase "lead to a significant increase" on Line 305 has been corrected to "leading to a significant increase".

11. Line 341, "withour" should be corrected to "with our".

**Reply:** Thank you for your advice. The typo "withour" on Line 341 has been corrected to "with our".

12. Line 380, "the horizontal gene transfer via natural transformation between nonpathogen is still high" should be changed to "the horizontal gene transfer via natural transformation among non-pathogens remains high".

**Reply:** Thank you for your advice. The sentence on Line 380 has been revised as suggested.

13. Line 382, "implementing measure" should be changed to "implementing measures".

**Reply:** The phrase "implementing measure" on Line 382 has been corrected to "implementing measures".

Once again, we thank you for your valuable feedback. We believe that these revisions have significantly improved the manuscript and hope that it is now suitable for publication in the journal.

Best Regards,

Xuan Gao
